# EVIDENCE-BASED REVIEWS & RECOMMENDATIONS FROM THE BRAZILIAN SOCIETY OF SHOULDER AND ELBOW SURGERY: REVERSE ARTHROPLASTY FOR COMPLEX PROXIMAL HUMERAL FRACTURES IN ELDERLY PATIENTS

**DOI:** 10.1590/1413-785220263401e303258

**Published:** 2026-08-31

**Authors:** Marcus Vinícius Galvão Amaral, Leonardo Zanesco, Gustavo de Mello Ribeiro Pinto, Maurício de Paiva Rafaelli, Thiago Barbosa Caixeta, Paulo Cesar Faiadi Piluski, Marcelo Costa de Oliveira Campos, Eduardo Angeli Malavolta

**Affiliations:** 1 Instituto Nacional de Traumatologia e Ortopedia Jamil Haddad, Centro de Cirurgia do Ombro e Cotovelo, Rio de Janeiro, RJ, Brazil.; 2 Universidade de Sao Paulo, Faculdade de Medicina, Hospital das Clinicas HCFMUSP, Sao Paulo, SP, Brazil.; 3 Hcor, Sao Paulo, SP, Brazil.; 4 Universidade do Estado do Rio de Janeiro (UERJ), Rio de Janeiro, RJ, Brazil.; 5 Instituto Naeon – Nucleo Avancado de Estudos em Ortopedia e Neurocirurgia, Sao Paulo, SP, Brazil.; 6 Universidade Federal de Goias, Goiania, Goias, GO, Brazil.; 7 Hospital Sao Vicente de Paulo, Instituto de Ortopedia e Traumatologia, Passo Fundo, RS, Brazil.

**Keywords:** Humeral Fractures, Arthroplasty, Replacement, Shoulder, Reverse Shoulder Arthroplasty, Fracture Fixation, Internal, Treatment Outcome, Fraturas do Úmero, Artroplastia do Ombro, Artroplastia Reversa do Ombro, Fixação Interna de Fraturas, Resultado do Tratamento

## Abstract

**Introduction:**

Complex (Neer 3- or 4-part) proximal humeral fractures in elderly patients are associated with high complication and failure rates after open reduction and internal fixation with locking plates (ORIF-LP). Reverse shoulder arthroplasty (RSA) has emerged as an alternative, but its comparative effectiveness remains debated.

**Methods:**

We conducted an evidence-based review of systematic reviews and randomized controlled trials including elderly patients with Neer 3- or 4-part fractures, identified through PubMed, Embase, Cochrane Library, and BVS (search to August 2025). High-quality systematic reviews were prioritized, and individual RCTs were added when necessary. Risk of bias and certainty of evidence were assessed using Cochrane methods and the GRADE approach.

**Results:**

Four systematic reviews and three RCTs met the inclusion criteria. Across network meta-analyses and direct comparisons, RSA provided superior shoulder function, with higher Constant and Oxford scores at 2 and 5 years than ORIF-LP. RSA also tended to reduce complication and reoperation rates, although event numbers were small and confidence intervals were wide. Overall certainty was low for function and very low for complications and reoperations.

**Conclusions:**

In elderly patients with complex proximal humeral fractures requiring surgery, RSA appears to yield better functional outcomes and fewer reoperations than ORIF-LP, supporting its use as the preferred surgical option, while acknowledging the low certainty of evidence. ***Level of evidence II; Systematic review.***

## RESUMO

**Introdução:**

Fraturas proximais complexas do úmero (Neer em 3 ou 4 partes) em pacientes idosos estão associadas a altas taxas de complicações e de falhas após redução aberta e fixação interna com placas bloqueadas (RAFI-PB). A artroplastia reversa do ombro (ARO) surgiu como alternativa, mas sua efetividade comparativa ainda é motivo de debate.

**Métodos:**

Foi realizada uma revisão baseada em evidências de revisões sistemáticas e ensaios clínicos randomizados que incluíram idosos com fraturas Neer em 3 ou 4 partes, identificados por meio das bases PubMed, Embase, Cochrane Library e BVS (busca até agosto de 2025). Revisões sistemáticas de alta qualidade foram priorizadas, e ensaios clínicos randomizados individuais foram acrescentados quando necessário. O risco de viés e a certeza da evidência foram avaliados segundo a metodologia Cochrane e a abordagem GRADE.

**Resultados:**

Quatro revisões sistemáticas e três ensaios clínicos randomizados preencheram os critérios de inclusão. Em meta-análises em rede e comparações diretas, a ARO apresentou melhor função do ombro, com escores de Constant e de Oxford superiores aos da RAFI-PB aos 2 e 5 anos. A ARO também tendeu a reduzir complicações e reoperações, embora com poucos eventos e amplos intervalos de confiança. A certeza global foi baixa para função e muito baixa para complicações e reoperações.

**Conclusão:**

Em idosos com fraturas proximais complexas do úmero que requerem tratamento cirúrgico, a ARO parece proporcionar melhores desfechos funcionais e menos reoperações do que a RAFI-PB, sustentando seu uso como opção cirúrgica preferencial, apesar da baixa certeza da evidência disponível. ***Nível de Evidência II; Revisão sistemática.***

## RECOMMENDATION

There is low-quality evidence showing that reverse shoulder arthroplasty (RSA) is more effective than open reduction and internal fixation (ORIF) with locking plates and screws for the treatment of complex proximal humeral fractures (defined as Neer 3- or 4-part fractures) regarding functional outcomes (Level of Evidence I and II).

There is also very low-quality evidence suggesting that RSA is more effective than ORIF with plates and screws in reducing complications and reoperation rates (Level of Evidence I and II).

The SBCOC Consensus Project recommends that elderly patients diagnosed with complex proximal humeral fractures (Neer 3 or 4 parts) should be individually evaluated, considering both clinical and injury characteristics, to determine the most appropriate treatment method. When surgery is chosen as the treatment approach, reverse shoulder arthroplasty should be regarded as the preferred surgical option, since it provides better functional outcomes, fewer complications, and lower reoperation rates, according to the current evidence.

## INTRODUCTION

Proximal humeral fractures (PHF) are highly prevalent, accounting for approximately 6% of all fractures,^1^ and mainly affect elderly patients.^2^

Although nonoperative management is frequently recommended in elderly patients^3^, some studies suggest that, based on clinical and radiographic criteria, certain fractures should be treated surgically.^4,5^

Among the surgical options, open reduction and internal fixation with locking plates and screws (ORIF-LP) has been widely used for the treatment of PHF.^6^ However, this technique is associated with a high complication rate, including intra-articular screw penetration, loss of reduction, avascular necrosis, malunion, nonunion, and infection.^7^

These complications are more common in elderly patients, resulting in revision rates of up to 29%.^8^ As an alternative, reverse shoulder arthroplasty (RSA) has emerged as a treatment option for complex PHF.^9^

Therefore, comparative studies between these surgical approaches, in terms of functional outcomes, complication rates, and reoperations, are crucial for optimizing management in elderly patients with complex PHF.

This evidence review was developed within a collaborative initiative between Cochrane and the Brazilian Society of Shoulder and Elbow Surgery (SBCOC). The aim of this partnership is to synthesize the best available evidence using rigorous, transparent methods and to translate it into clear, practical recommendations for clinical practice.

### Research Question

In elderly patients with complex proximal humeral fractures, which treatment is more effective — reverse shoulder arthroplasty (RSA) or open reduction and internal fixation with locking plates and screws (ORIF-LP) — regarding functional outcomes, complication rates, and reoperation needs?

## METHODS

- Databases searched: PubMed, Embase, Cochrane Library, and BVS.
- Descriptors used:
- "Humeral Fractures" [Mesh]; " Reverse Shoulder Arthroplasty" OR " Arthroplasty, Replacement, Shoulder"[Mesh]; "Fracture Fixation, Internal "[Mesh]; " Systematic Review" [Publication Type]; " Randomized Controlled Trial" [Publication Type] (see Appendix 1).
- Search period: Up to August 2025.
- Systematic reviews (SRs) with high methodological quality and recent publication were prioritized. In the absence of high-quality SRs, randomized controlled trials (RCTs) with adequate methodological quality were included.

## RESULTS

The search identified 100 references (40 PubMed, 36 Cochrane Library, 13 Embase, and 11 from the Regional BVS Portal). Among these, 14 were systematic reviews and 3 were randomized controlled trials.

After full-text analysis, 10 systematic reviews^9–18^ were excluded for addressing 2-part fractures or for not focusing exclusively on elderly patients, and one systematic review^19^ was excluded due to an updated version.

**Figure 1:**
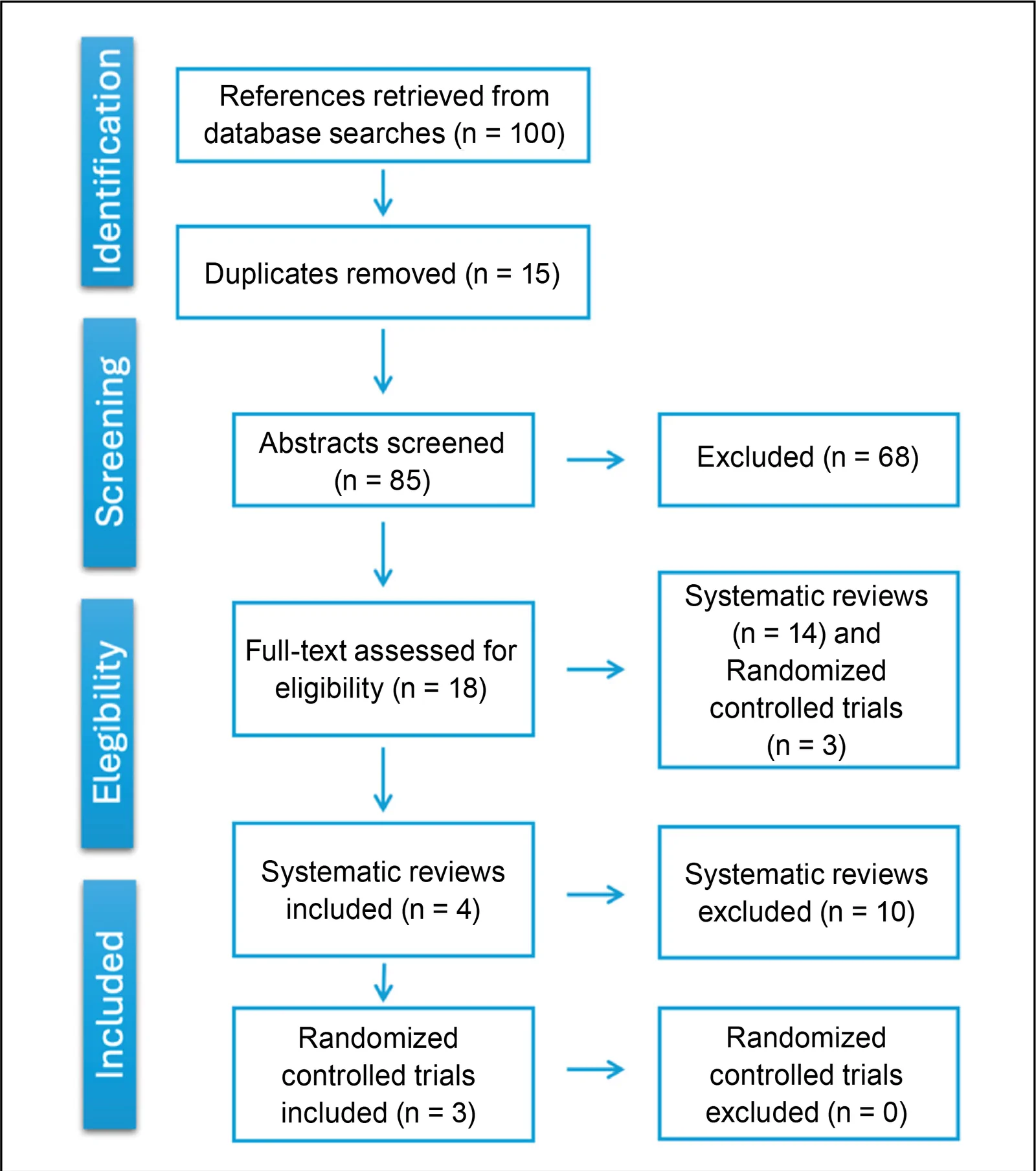
Flow diagram of the literature search.

Thus, 4 systematic reviews^20–23^ and 3 randomized controlled trials^24–26^ were included in the final synthesis. One of these RCTs reported outcomes from the same sample at 5-year follow-up.^26^ (Figure 1).

### Included studies

The first review addressing the clinical question was conducted by Du et al.^20^ (2017), which compared interventions for the treatment of 3- and 4-part proximal humeral fractures (PHF) in elderly patients.

A total of 7 randomized controlled trials (RCTs) with 347 participants were included, comparing nonoperative treatment with sling (108 participants), hemiarthroplasty (93 participants), open reduction and internal fixation with locking plate (ORIF-LP; 76 participants), and reverse shoulder arthroplasty (RSA; 31 participants). None of the studies included a direct comparison between ORIF-LP and RSA. The authors performed a network meta-analysis to compare these interventions, using Constant scores and reoperation rates as primary outcomes, analyzed with Cochrane methodology.^27^ They concluded that, for both functional outcomes and reoperation rates, RSA showed superior results compared with ORIF-LP.

Guo et al.^23^ (2022) also conducted a network meta-analysis including only RCTs. Their comparisons were similar to those in Du et al.^20^, but an additional group treated with intramedullary nailing was analyzed. Outcomes included the Constant score and reoperation rates. A total of 11 studies with 648 participants were included, and only one study directly compared RSA with ORIF-LP. According to the authors, all included studies presented a low risk of bias, as assessed by the Cochrane tool. The authors concluded that RSA was the most effective intervention, demonstrating lower reoperation rates. In both Du et al.^20^ and Guo et al.,^23^ the follow-up period was less than 2 years.

Two additional systematic reviews compared ORIF-LP and RSA — one published in 2022^21^ and the other in 2023.^22^ However, like the previous review,^23^ both included only one RCT^24^ for data extraction and analysis.

That Fraser et al.^24^ study was a multicenter RCT conducted in Norway, including 124 participants aged 65–85 years with complex PHF, recruited from seven public hospitals. A research protocol had been previously published.^29^ Outcomes of interest included shoulder function (Constant and Oxford Shoulder Scores), adverse events, and revision surgery rates. The primary endpoint was the Constant score at 2 years, showing that patients who underwent RSA achieved a mean score of 68 points (95% CI, 63.7–72.4), whereas the ORIF-LP group achieved 54.6 (95% CI, 48.5–60.7) — a mean difference of 13.4 points (95% CI, 6.2–20.6; p < 0.001), which was both statistically and clinically significant in favor of RSA.

For the Oxford Shoulder Score, RSA patients achieved a mean of 40.8 (95% CI, 38.8–42.7), while the ORIF-LP group averaged 36.5 (95% CI, 34.0–39.0), corresponding to a mean difference of 4.3 points (95% CI, 1.2–7.4; p = 0.007), again favoring RSA.

Regarding complications, there were 7 events among 7 RSA patients versus 12 events among 11 ORIF-LP patients. Of these, reoperations occurred in 4 RSA patients (2 component exchanges, 2 others) and 8 ORIF-LP patients (4 conversions to RSA and 4 hardware removals or ORIF revisions). In a 2024 follow-up publication,^25^ the same cohort was reassessed at 5 years postoperatively. Follow-up was available for 65 of the 124 patients, representing a substantial attrition that limits statistical power and generalizability. The mean Constant score was 71.7 (95% CI, 67.1–76.3) for RSA and 58.3 (95% CI, 50.6–65.9) for ORIF-LP, with a mean difference of 13.4 points (95% CI, 5.2–21.7; p < 0.002) in favor of RSA. The Oxford Shoulder Score averaged 43.0 (95% CI, 41.3–44.8) in the RSA group and 38.0 (95% CI, 34.8–41.3) in the ORIF-LP group, a mean difference of 5.0 points (95% CI, 1.7–8.3; p = 0.003), also favoring RSA. Over the 5-year period, 13 complications occurred among RSA patients and 17 among ORIF-LP patients. There were 6 reoperations in the RSA group and 11 in the ORIF-LP group.

Both studies concluded that RSA is superior to ORIF-LP for elderly patients regarding shoulder function, adverse events, and reoperation rates.

A recent publication by Yang et al.26 (2025) — not previously included in earlier systematic reviews — compared these two treatments in 68 patients aged ≥70 years with complex PHF. All surgeries were performed by the same surgeon. Outcomes included Constant and Oxford Shoulder Scores at 2 years, as well as complication and reoperation rates. The mean improvement in Constant score was 30.3 ± 8 for RSA and 22.6 ± 6.0 for ORIF-LP, with a mean difference of 7.7 points (95% CI, 4.0–11.3; p < 0.001). For the Oxford Shoulder Score, RSA patients scored 29.5 ± 4.3, while ORIF-LP patients scored 27.2 ± 4.4, a mean difference of 2.3 (95% CI, 0.2–4.4; p = 0.032). Complication rates did not differ significantly (RSA 9.4% [3/32] vs. ORIF-LP 16.7% [6/36]; OR = 0.828 [95% CI 0.171–4.014]; p = 0.814). However, no RSA patients required reoperation, whereas four ORIF-LP patients underwent additional surgery.

The general characteristics of the evaluated studies and the quality of evidence according to the GRADE system^30,31^ are presented in Tables 1 and 2, respectively.

**Table 1:** Characteristics of the included studies.

Studies	Study type	Included studies (Designs)	Sample size	Published protocol
Du et al. (2017)	Systematic review	7 (RCTs)	347	No
Guo et al. (2023)	Randomized control trial (RCT)	11 (RCTs)	648	Yes
Handol et al. (2022)	Randomized control trial (RCT)	47 (RCTs)	3179	No
Lapner et al. (2023)	Randomized control trial (RCT)	15 (RCTs)	1146	Yes

**Table 2:** Quality of evidence (GRADE).

Outcome	Studies (n)	Evidence quality
Function (Constant score)	2 studies	Low
Complication rate	2 studies	Very low
Reoperation rate	2 studies	Very low

The GRADE tool (Grading of Recommendations Assessment, Development and Evaluation) is used to appraise and measure the quality of evidence. It is grounded in five domains: risk of bias, imprecision, inconsistency, indirectness of outcomes, and publication bias. Based on this framework, the quality of evidence is classified into four levels: very low, low, moderate, and high.^30,31^

## CONCLUSION

For the outcome of shoulder function (measured by the Constant score), RSA is superior to ORIF-LP in the treatment of Neer 3- or 4-part proximal humeral fractures in elderly patients, with a low level of evidence. For the outcomes of complication rate and reoperation rate, RSA also shows better results than ORIF-LP, though the evidence quality is very low.

## Data Availability

The data underlying the research text are contained in the manuscript.

## Appendix 1 Database search strategies.

**Table:**

**PUBMED**
#1 "Humeral Fractures"[Mesh] OR (Fracture, Humeral) OR (Humeral Fracture) OR (Humeri Fractures) OR (Fracture, Humeri) OR (Humeri Fracture) OR (Humerus Fractures) OR (Fracture, Humerus) OR (Humerus Fracture)
#2 (Reverse Shoulder Arthroplasty) OR "Arthroplasty, Replacement, Shoulder"[Mesh] OR (Shoulder Replacement Arthroplasty) OR (Arthroplasties, Shoulder Replacement) OR (Arthroplasty, Shoulder Replacement) OR (Replacement Arthroplasties, Shoulder) OR (Replacement Arthroplasty, Shoulder) OR (Shoulder Replacement Arthroplasties) OR (Total Shoulder Replacement) OR (Replacements, Total Shoulder) OR (Replacement, Total Shoulder) OR (Shoulder Replacements, Total) OR (Shoulder Replacement, Total) OR (Total Shoulder Replacements)
#3 "Fracture Fixation, Internal"[Mesh] OR (Fixation, Internal Fracture) OR (Fixations, Internal Fracture) OR (Fracture Fixations, Internal) OR (Internal Fracture Fixation) OR (Internal Fracture Fixations) OR (Osteosynthesis, Fracture) OR (Fracture Osteosyntheses) OR (Fracture Osteosynthesis) OR (Osteosyntheses, Fracture)
#4 "Bone Plates"[Mesh] OR (Bone Plate) OR (Plate, Bone) OR (Plates, Bone)
#5 #3 OR #4
#6 #1 AND #2 AND #5
Filters applied: Randomized Controlled Trial, Systematic Review

**Table:**

**COCHRANE**
#1 (Humeral Fractures) OR (Fracture, Humeral) OR (Humeral Fracture) OR (Humeri Fractures) OR (Fracture, Humeri) OR (Humeri Fracture) OR (Humerus Fractures) OR (Fracture, Humerus) OR (Humerus Fracture)
#2 (Reverse Shoulder Arthroplasty) OR (Arthroplasty, Replacement, Shoulder) OR (Shoulder Replacement Arthroplasty) OR (Arthroplasties, Shoulder Replacement) OR (Arthroplasty, Shoulder Replacement) OR (Replacement Arthroplasties, Shoulder) OR (Replacement Arthroplasty, Shoulder) OR (Shoulder Replacement Arthroplasties) OR (Total Shoulder Replacement) OR (Replacements, Total Shoulder) OR (Replacement, Total Shoulder) OR (Shoulder Replacements, Total) OR (Shoulder Replacement, Total) OR (Total Shoulder Replacements)
#3 (Fracture Fixation, Internal) OR (Fixation, Internal Fracture) OR (Fixations, Internal Fracture) OR (Fracture Fixations, Internal) OR (Internal Fracture Fixation) OR (Internal Fracture Fixations) OR (Osteosynthesis, Fracture) OR (Fracture Osteosyntheses) OR (Fracture Osteosynthesis) OR (Osteosyntheses, Fracture)
#4 (Bone Plates) OR (Bone Plate) OR (Plate, Bone) OR (Plates, Bone)
#5 #3 OR #4
#6 #1 AND #2 AND #5

**Table:**

**EMBASE**
#1 ‘humerus fracture’/exp OR ‘fracture, humerus’ OR ‘humeral fracture’ OR ‘humeral fractures’ OR ‘humerus fracture’ OR ‘humerus juxtaarticular fracture’ OR ‘humerus medial condyle fracture’ OR ‘humerus medial epicondyle fracture’ OR ‘upper arm fracture’
#2 ‘shoulder replacement’/exp OR ‘arthroplasty, replacement, shoulder’ OR ‘shoulder replacement’ OR ‘shoulder replacement arthroplasty’ OR ‘shoulder replacements’ OR (Reverse Shoulder Arthroplasty)
#3 ‘osteosynthesis’/exp OR ‘fracture fixation, internal’ OR ‘gleitosteosynthesis’ OR ‘internal fixation’ OR ‘internal fracture fixation’ OR ‘osteo synthesis’ OR ‘osteosynthesis’
#4 ‘bone plate’/exp OR ‘claw ii’ OR ‘integra (bone plate)’ OR ‘lcp (bone plate)’ OR ‘lcp distal ulna plate’ OR ‘marc k (bone plate)’ OR ‘ncb (bone plate)’ OR ‘pediatric lcp’ OR ‘rival reduce’ OR ‘rival view’ OR ‘sutureplate’ OR ‘tbfix’ OR ‘trumatch (bone plate)’ OR ‘valkyrie’ OR ‘visiofix’ OR ‘bone fixation plate’ OR ‘bone plate’ OR ‘bone plate, device’ OR ‘bone plates’ OR ‘fixation plate’ OR ‘fracture fixation plate’ OR ‘growth-correction orthopaedic fixation plate kit’ OR ‘growth-correction orthopedic fixation plate kit’ OR ‘non bioabsorbable orthopedic fixation plate’ OR ‘non-bioabsorbable bone plate’ OR ‘non-bioabsorbable fixation plate’ OR ‘non-bioabsorbable orthopaedic fixation plate’ OR ‘nonsterile non-bioabsorbable orthopaedic fixation plate’ OR ‘non-sterile nonbioabsorbable orthopedic fixation plate’ OR ‘orthopaedic fixation plate, non-bioabsorbable, non-sterile’ OR ‘orthopaedic fixation plate, non-bioabsorbable, sterile’ OR ‘orthopaedic fixation plates’ OR ‘orthopedic fixation plate, non bioabsorbable’ OR ‘orthopedic fixation plate, nonbioabsorbable, non-sterile’ OR ‘orthopedic fixation plates’ OR ‘rhead’ OR ‘semitubular plate’ OR ‘sterile non-bioabsorbable orthopaedic fixation plate’ OR ‘tubular plate’
#5 #3 OR #4
#6 #1 AND #2 AND #5
#7 AND [embase]/lim NOT ([embase]/lim AND [medline]/lim)
#8 AND [embase]/lim NOT ([embase]/lim AND [medline]/lim) AND ([cochrane review]/lim OR [systematic review]/lim OR [meta analysis]/lim OR [controlled clinical trial]/lim OR [randomized controlled trial]/lim)

**Table:**

**PORTAL REGIONAL BVS**
#1 MH:"Fraturas do Úmero" OR (Humeral Fractures) OR (Fratura do Úmero) OR (Fraturas dos Úmeros) OR MH:C26.088.390$ OR MH:C26.404.500$
#2 MH:"Artroplastia do Ombro" OR (Arthroplasty, Replacement, Shoulder) OR (Substituição Total do Ombro) OR MH:E04.555.110.110.299$ OR MH:E04.617.101.110.299$ OR MH:E04.650.110.299$
#3 MH:"Fixação Interna de Fraturas" OR (Fracture Fixation, Internal) OR (Osteossíntese) OR (Osteossíntese em Fratura Cirúrgica) OR MH:E02.718.472.300$ OR MH:E04.555.300.300$
#4 MH:"Placas Ósseas" OR (Bone Plates) OR (Placas Ortopédicas) OR MH:E07.695.370.374$ OR MH:E07.858.442.660.460.374$ OR MH:E07.858.690.725.460.374$
#5 #3 OR #4
#6 #1 AND #2
